# Dystrophin gene editing by CRISPR/Cas9 system in human skeletal muscle cell line (HSkMC)

**DOI:** 10.22038/IJBMS.2021.54711.12269

**Published:** 2021-08

**Authors:** Mahintaj Dara, Vahid Razban, Mohsen Mazloomrezaei, Maryam Ranjbar, Marjan Nourigorji, Mehdi Dianatpour

**Affiliations:** 1 Department of Molecular Medicine, School of Advanced Medical Science and Technology, Shiraz University of Medical Science, Shiraz, Iran; 2 Student Research Committee, Shiraz University of Medical Science, Shiraz, Iran; 3 Department of Medical Genetics, School of Medicine, Shiraz University of Medical Sciences, Shiraz, Iran; 4 Stem Cell Technology Research Center, Shiraz University of Medical Sciences, Shiraz, Iran

**Keywords:** CRISPR/Cas9, DMD, Dystrophin, Gene editing, HSkMC

## Abstract

**Objective(s):**

Duchene muscular dystrophy (DMD) is a progressive neuromuscular disease caused by mutations in the *DMD* gene, resulting in the absence of dystrophin expression leading to membrane fragility and myofibril necrosis in the muscle cells. Because of progressive weakness in the skeletal and cardiac muscles, premature death is inevitable. There is no curative treatment available for DMD. In recent years, advances in genetic engineering tools have made it possible to manipulate gene sequences and accurately modify disease-causing mutations. CRISPR/Cas9 technology is a promising tool for gene editing because of its ability to induce double-strand breaks in the DNA.

**Materials and Methods:**

In this study for the exon-skipping approach, we designed a new pair of guide RNAs (gRNA) to induce large deletion of exons 48 to 53 in the *DMD* gene in the human skeletal muscle cell line (HSkMC), in order to correct the frame of the gene.

**Results:**

Data showed successful editing of *DMD* gene by deletion of exons 48 to 53 and correction of the reading frame in edited cells. Despite a large deletion in the edited *DMD* gene, the data of real-time PCR, immune florescent staining demonstrated successful expression of truncated dystrophin in edited cells.

**Conclusion:**

This study demonstrated that the removal of exons 48-53 by the CRISPR / Cas9 system did not alter the expression of the *DMD* gene due to the preservation of the reading frame of the gene.

## Introduction

Duchene muscular dystrophy (DMD) is one of the most common lethal neuromuscular disorders that approximately affects 1 in 3500 to 5000 male births. The inheritance pattern of DMD is X-linked recessive (1, 2). DMD is mainly caused by a frame-shift mutation in the 2.2 megabase pair *DMD* gene, which prevents expression of the dystrophin protein (3). Dystrophin is a member of the dystrophin-glycoprotein complex (DGC) and has an essential role in the stability of sarcolemma by connecting the actin cytoskeleton to the extracellular matrix (4). Disruption of DGC in the lack of dystrophin causes damage to muscle fibers resulting in progressive muscle degeneration leading to heart and respiratory failure and premature death (5, 6). Although all 79 exons of the* DMD* gene can bear mutation, exons 45 to 55 are mutational hotspot regions, which contain more than 60% of deleterious mutations (7). Different types of mutations have been described in DMD, the most common of these are large deletions and duplications, and small point mutations like frameshift, missense, and nonsense mutations consist of the rest of the mutations (8-10).

In contrast to DMD, Becker muscular dystrophy (BMD) is caused by in-frame mutations in dystrophin encoding gene- *DMD* - resulting in the expression of functional truncated dystrophin; therefore, BMD patients show the moderate form of disease with late-onset muscular dystrophy (11). At present, there is no effective treatment for DMD and common approaches are typically palliative and supportive (12). Recently, some proposed treatments for DMD have focused on modulating the disease and inducing symptoms similar to BMD. Exon skipping strategy by antisense oligonucleotide and delivery of truncated dystrophin such as mini- and micro-dystrophin are some of these approaches (13-16).

On the other hand, emerging gene-editing technologies such as ZFN (Zinc finger nuclease), TALEN (transcription activator-like effectors), and CRISPR (clustered regularly interspaced short palindromic repeats) present a promising approach toward editing and treating genetic disorders like DMD (17-20). CRISPR-Cas9 is a bacterial **adaptive **immune system that has been optimized as a powerful gene-editing technology (21, 22). This tool consists of a short guide RNA (sgRNA) which guides Cas9 nuclease to a specific genome sequence near a protospacer adjacent motif (PAM) and induces double-strand breaks (DSB) in DNA (23-25). Consequently, DSBs trigger endogenous DNA repair pathways: NHEJ (non-homologous end joining) or HDR (homology-directed repair) (26-28). We aimed to utilize CRISPR/Cas9 to efficiently edit the dystrophin gene and compare the expression of the edited dystrophin *vs* no edited dystrophin in the human skeletal muscle cell line. In this study we unlike others deleted 6 exons of dystrophin gene exons from 48 to exon 53, this part of the *DMD* gene is the most prevalent site for deletion. Using these sgRNAs enables us to edit a broad range of deletions. The cell line that we used in this study was the human skeletal muscle cell line that is myoblast cell and suitable for dystrophin gene expression and dystrophin gene editing. To the best of our knowledge, this is the first study using this cell line for dystrophin gene editing.

## Materials and Methods

***gRNA design and cloning***

**Figure 1:**
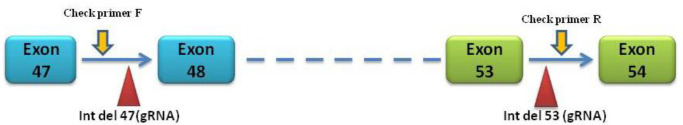
Schematic construct map and primer site according to our design for edition of dystrophin

According to the *DMD* gene sequence (NG_012232.1 RefSeqGene, NCBI), gRNAs were designed by https://zlab.bio/guide-design-resources and validated using a Cas-OFFinder (www.rgenome.net/cas-offinder/)(Figure 1). We chose a pair of gRNAs, intDel 47 and intDel 53 to conduct further experiments in the human skeletal muscle cell line (HSkMC). The gRNAs were cloned into pSpCas9 (BB)-2A-GFP (PX458) (Cat No.48138.addgene) using the BbsI enzyme (Thermo Scientific). Selected gRNA sequences were presented in Table 1. The plasmid constructs were transformed into the *DH5a Escherichia coli *and extracted using the Miniprep kit (Qiagen. USA).

***Cell culture***

Human embryonic kidney-293 cells (ATCC: CRL-1573) were cultured in F12 DMEM (Dulbecco’s Modified Eagle’s Medium) media that was supplemented with 10% FBS (fetal bovine serum) (Gibco) and 1% pen-strep (penicillin-streptomycin) (Thermo Fisher Scientific). After 2 subcultures, the cells were transferred to the 6 well plates and seeded with Opti-mem media (Reduced Serum Media Thermo Fisher Scientific); after 24 hr, the cloned vector was transfected to the cells (with 60% confluency) by lipofectamine 2000 (Invitrogen) according to the manufacturer’s instructions. After 8 hr of transfection, Opti-mem media were discarded and complete DMEM media containing 10% FBS and 1% pen-strep were added to the cells. After 48 to 72 hr, the cells were observed by fluorescence microscopy.

The human skeletal muscle cell line (HSkMC) (NCBI code: C598) was purchased from the Pasteur Institute of Iran. HSCkM cells were cultured in DMEM high glucose supplemented with 10% FBS, 1% penicillin/streptomycin, and 5 µg/ml basic fibroblast growth factor (bFGF). Cells were incubated at 37 °C, 5% CO_2_. One day before transfection, the cells were seeded into the 6-well plates and cultured with F12 media, supplemented by 5% FBS. Due to the low transfection efficiency of lipofectamine in the human skeletal muscle cells, we used another transfection agent, TRANSFECTimin (Dara Zistfan Eram, Iran ). HSkMC were transfected with 10 μg of the plasmid constructs by transfection agent TRANSFECTimin. To compare the expression of edited (truncated) dystrophin with the wild type, we transfected the HSkMC cells by 3 different strategies, that were named according to the following code: eHSkMC (edited HSkMC transfected by intDel47 and intDel53), bHSkMC (HSkMC transfected by empty backbone PX458) and wHSkMC (nontransfected or wild type). 

***Fluorescence-activated cell sorting and single-cell isolation***

Two days after transfection, the cells were trypsinized and collected for Fluorescence-Activated Cell Sorting and single-cell isolation using FACS AryaIII instrument (BD Bioscience). GFP-positive single cells were collected and expanded for the following molecular analysis.

***Genomic DNA extraction and PCR-base assay***

To detect the induced deletion in edited cells, we used primers for the flanking region of the deleted area, “check F” and “check R” (Table 2). Genomic DNA was extracted (DNA extraction Kit QIAGEN, USA) and PCR was performed by the following program: 95 °C for 2 min, 30 cycles (95 °C for 30 sec, 58 °C for 30 sec, and 72 °C for 40 sec), 72 °C for 5 min; followed by holding at 4 °C.

***mRNA analysis and real-time PCR***

The total RNA was extracted from HSkMC cells using the RNeasy Plus Mini Kit (QIAGEN, USA) according to the manufacturer’s instructions. Reverse transcription of 1 µg total RNA was carried out by cDNA synthesis (Takara, Japan). cDNA was synthesized in the following conditions according to the instructions: 15 min at 37 °C and 5 sec at 85 °C. Two sets of primers were designed; one pair contained forward primer 15-F (complementary to exon 15) and reverse primer 16-R (reverse complementary to exon 16) as control of dystrophin expression in both edited and non-edited cells, and the other pairs consisted of 47-F (complementary to exon 47) and 48-R (reverse complementary to exon 48) (Table 3). Amplification was done in an Applied Biosystem 7500 Real-Time PCR system.

***Immune florescent staining***

To analyze dystrophin expression in the edited cells, the cells were coated on glass slides and fixed with 4% PFA (paraformaldehyde)(Sigma) for 5 min at room temperature. After permeabilization using 0.2% Triton X-100, the samples were washed twice with cold TPBS and incubated with a blocking solution containing 1% BSA in PBS for 1 hr at room temperature. The cells were stained using a primary antibody (ab15277; Abcam) overnight at 4 °C and then washed by cold TPBS and incubated with a secondary antibody (IgG-FITC-SantaCruz) for 1 hr at room temperature. Nucleic acids were labeled with DAPI. 

## Results

**Figure 2:**
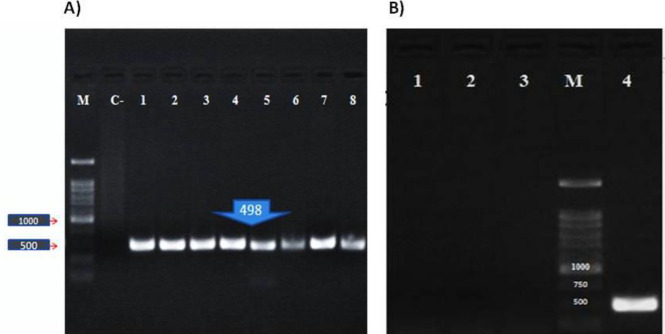
Detection of the edited cells by PCR. A) Amplification of 498bp in edited cells. M (DNA Marker 1kb, Cinnagen), C-(negative control or non-edited HEK-293 cells), 1-8 (edited HEK-293 cells). B) Negative control (1), w- HSkMC or no edited cells (2), b-HSkMC, cells infected with PX458 empty or backbone (3), Amplification of 498bp fragment in e-HSkMC or edited cells (4), and DNA marker 1kb (Cinnagen. Iran) (M)

**Figure 3:**
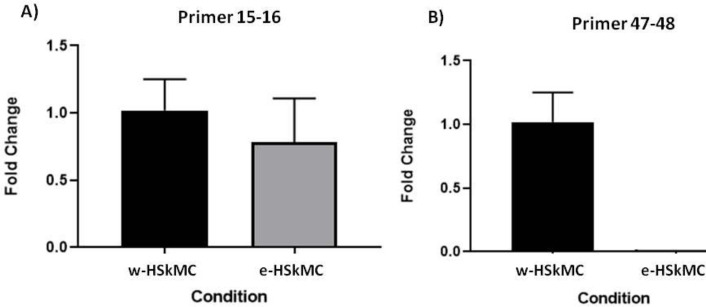
Dystrophin expression in the edited cells (e-HSkMC) and non-edited cells (w-HSkMC). GAPDH was used as an internal control gene. A) Plot demonstrates the total RNA expression in edited (e-HSkMC) and non-edited cells (w-HSkMC) checked by primer designed for exons 15 and 16. B) Plot demonstrated deletion of exons 48-53 in the edited cell, so amplification was not seen by primer for exons 47 and 48 in edited cells (*P*-value >0.05)

**Figure 4:**
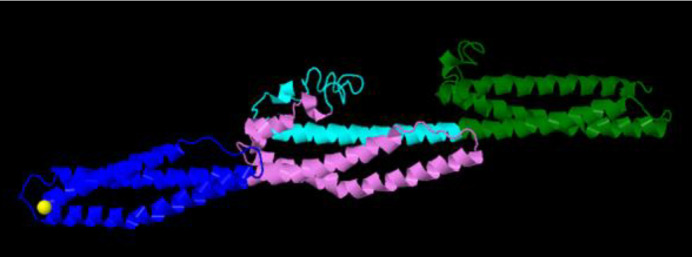
According to analysis of the *DMD* gene in www.edystrophin.genouest.org, deletion of exons 48-53 resulted in removal of a part of R18 to R 22 that is located in the rod domain. Rod domain of dystrophin is composed of 24 repeats (R1 to R24) homologue to spectrin repeats and 4 hinges. Deletion of exons 48-55 modeling from repeat 17 to repeat 23 from I-Tasser ( score: -0.94). Blue: R17, Violet: R18, Cyan: R22, Green: R23

**Figure 5:**
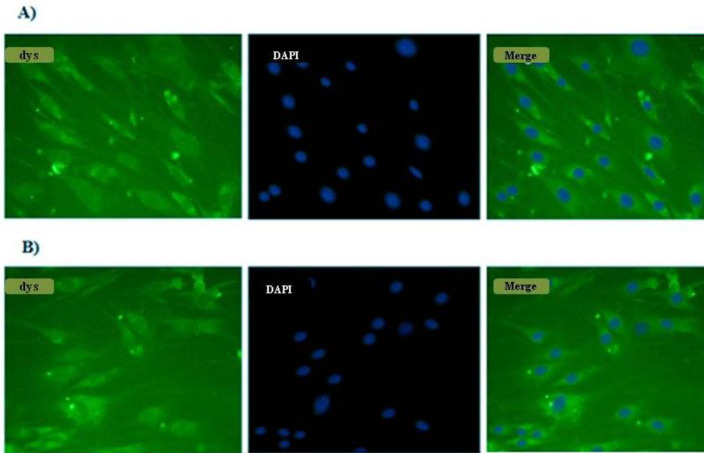
Immune fluorescent staining using dystrophin Ab and DAPI as counter-stain in edited cells (e-HSkMC) and non-edited cells (w-HSkMC). A) e-HSkMC. B) w-HSkMC. Both eHSkMC and wHSkMC showed a positive result in immune fluorescent staining using dystrophin Ab. These results demonstrate that dystrophin could express in the edited cells

According to our study design, we chose exons 48-53 located in the hotspot mutational region of the *DMD* gene. The edited cells showed deletion in exons 48 to 53 in the *DMD* gene and resulted in the attachment of exon 47 to exon 54 which produced a shortened in-frame dystrophin gene. Transfection efficiency was 70%. 72 hr after transfection, the total genomic DNA was isolated. We directly analyzed genomic DNA in the transfected and nontransfected cells. To do this, specific PCR was performed by a forward primer located in intron 47 (check F) and reverse primer (check R) located in intron 53. These primers resulted in amplification of a 498bp fragment in the edited cells; no amplification was seen in control cells (Figure 2). Direct sequencing of the 498bp amplicon indicated the deletion of exon 48 to 53. By performing agarose gel electrophoresis, a 500bp fragment was seen in eHSkMC, while we did not have any product in bHSkMC and wHSkMC (Figure 3). This result indicated correct deletion of exons 48 to 53 in eHSkMC in comparison with bHSkMC and wHSkMC. To study dystrophin expression in mRNA level, total RNA was extracted from eHSkMC, bHSkMC, and wHSkMC. After quantitative PCR, amplification was detected in edited (eHSkMC) and control (wHSkMC) cells by 15-16 F&R primers. According to the design of 47-48 primers (48-R reverse complementary to exon 48), if edition occurs in the cells, the reverse primer cannot anneal to the exon 48; therefore, amplification was not seen. As expected, amplification was detected only in wHSkMC, which indicated deletion of exon 48 to 53 in eHSkMC. After agarose gel electrophoresis, the amplicon was extracted and cloned in PMinT plasmid (data not shown) and sent for sequencing. mRNA analysis results demonstrated dystrophin expression in the edited and non-edited cells (Figure 3). Base on bioinformatics analysis (www.edystrophin.genouest.org), deletion of these exons results in the production of a truncated and partially functional protein with a size of 390 kDa which is 37 kDa shorter than wild dystrophin. Deletion of exons 48-55 was modeled using I-Tasser (Iterative Threading Assembly Refinement) from protein modeling databank. (Table 4, Figure 4). Immune fluorescent staining of dystrophin (Figure 5) in eHSkMC in comparison with wHSkMC showed that truncated dystrophin could be expressed in the edited cell.

**Table 1:** Selected gRNA for dystrophin gene edition

Sequence	Guides
F : 5’-CACCGAACTGCAAAGGAAGCGCGTA- 3’ R: 3’-CTTGACGTTTCCTTCGCGCATCAAA- 5’	**Int del 47**
F: 5’ –CACCGCCGCACATGGTGGTGCGGAC- 3’ R: 3’-CGGCGTGTACCACCACGCCTGCAAA -5’	**Int del 53**

**Table 2:** Primer set for detection of edition in dystrophin edited cells by CRISPR/Cas9

Product size	Sequence	Name
500bp	F:5-GACTCACAAACTATAGCTCACA-3’ R :5’-TCAAGGTAGAGAATAGAGG-3’	Check primer

**Table 3:** Real-time PCR primer for confirmation of correct edition

Size	Sequence	Name
141bp	F: 5’- AGATTCACACAACTGGCTT-3’ R: 5’- TTCTGGGTCACTGACTTATTC-3’	Primer 15-16
131bp	F: 5’-AGGACCCGTGCTTGTAAGTG-3’ R: 5’-AAGCTGCCCAAGGTCTTTTA-3’	Primer 47-48

**Table 4:** Comparison of length and size of wild dystrophin with edited dystrophin

	Full length dystrophin	Deletedexon 48-56^*^ dystrophin
Protein size	3685 aa	3365 aa
Protein weigh	427kDa	390 kDa

## Discussion

The dystrophin gene, which mainly expresses in the skeletal muscle cells, is a large gene in the human genome that spans about 2.2 Mbp, located on the X chromosome in the Xp21 region (29). Rod shaped dystrophin protein has four domains: N-terminal or actin-binding domain, central rod domain, cysteine-rich domain, and C-terminal domain. Two domains, N-terminal and C-terminal, have a crucial role in dystrophin function in connecting actin proteins to the cytoskeleton, thus stabilizing the sarcolemma (30-32). Mutation in the *DMD* gene causes progressive degeneration of the skeletal and cardiac muscles that leads to premature death due to cardiopulmonary complications (33-35). There is no definite treatment for DMD and current approaches including administration of corticosteroids and physical therapy are mostly supportive and alleviate the symptoms of the patients, but do not target the underlying cause of diseases (36, 37). Although the large size of the *DMD* gene and abundant affected tissues are two main obstacles in *DMD* gene therapy, gene therapy of DMD was more considered in the past decades (38). Two main approaches for gene therapy in DMD are delivery of engineered mini dystrophin (lacking non-essential domains) and up-regulation of utrophin that is one of the dystrophin isoforms (39, 40). Another strategy is the application of Antisense Oligo Nucleotides (AONs) which induce exon skipping that restores the reading frame of dystrophin transcript (41-43). AONs have shown promising results, but due to the transient nature of this treatment, patients should treat constantly during their lifetime (33). The use of RNA-DNA chimeric oligonucleotide (RDOs) or oligodeoxynucleotides (ODNs) is another strategy in DMD treatment (34, 35). Advancements in molecular technologies have paved the way for developing definite treatments for genetic diseases. Meganucleases, ZFNs, and TALENs were used for *DMD* gene editing *in vivo* and *in vitro*. They have achieved promising results but complicated the manufacturing procedures, and lower sequence target ability is the challenge yet to be addressed (44, 45). Recently, RNA-guided endonucleases, such as CRISPR/Cas9, which have several unique features in comparison with other gene-editing tools, have been considered more applicable for correcting genetic disorders (39, 40, 44).

Compared to other methods, the CRISPR cas9 construction technique is more straightforward, and gRNA could mediate cleavage in any region of the genome adjacent to a PAM sequence, providing targeted gene editing (46,47). Deletions, point mutations, and duplications occur frequently in the *DMD* gene. Considering the efficiency rate of CRISPR cas9, using a pair of gRNAs for editing of mutated exons is more preferred (48-50).

 In one study Tabebordbar* et al.*, deleted exon 23 in mouse muscle and stem cells of mdx mice by CRISPR/Cas9. Their results indicated restoration of dystrophin in the edited cells (35). In another study *Young et al.*, targeted exons 45 to 55 in DMD patient derived hiPSCs by CRISPR/Cas9. In this study, a 750 kb fragment was deleted in *the DMD* gene in order to produce an in-frame mutation by which dystrophin expression was restored in the edited cells (37). Min* et al.* deleted exon 44 in cardiomyocytes differentiated from hiPSCs cells by CRISPR/Cas9 and restored dystrophin expression in the edited cells (36). In the present study, we targeted exons 48 to 53 that are located in the hotspot mutational region of the* DMD* gene, using the CRISPR/Cas9 system, and analyzed the expression of the truncated dystrophin in comparison with the wild type dystrophin in the human skeletal muscle cells. Our designed gRNAs could successfully delete exons 48-53 and acted as universal gRNA to target approximately 60% of mutations in the *DMD* gene. Moreover, we directly used human skeletal muscles cells rather than mouse muscle cells or hi-PSCs. Functional validation of truncated dystrophin would require *in vivo* studies. Further *in vivo* studies on the efficacy of the CRISPR cas9 gene-editing tool are needed in order to use this method as a promising treatment for DMD in the near future.

## Conclusion

Our result indicates that successful deletion of exon 48 to 53, leads to expression of truncated dystrophin in the edited cells. A pair of designed gRNAs in this study was selected using molecular assays to be used as universal gRNAs for editing any type of mutation that could occur in this particular hotspot region. Finally, this study demonstrated that the removal of exons 48-53 by the CRISPR / Cas9 system did not alter the expression of the Dmd gene due to the preservation of the reading frame of the gene.
